# The Role of Relationship Quality and Perceived Partner Responses with Pain and Disability in Those with Back Pain

**DOI:** 10.1111/j.1526-4637.2011.01298.x

**Published:** 2012-01-05

**Authors:** Paul Campbell, Kelvin P Jordan, Kate M Dunn

**Affiliations:** Arthritis Research UK Primary Care Centre, Keele UniversityUK

**Keywords:** Low Back Pain, Relationship Quality, Spouse Responses, Depression, Biopsychosocial

## Abstract

**Objective:**

The objectives of this study were to investigate the associations of key constructs of relationship quality (cohesion, consensus, and satisfaction) and perceived partner responses to pain behavior (e.g., solicitous and negative responses) with the outcomes of pain and disability in those with long-term low back pain, and to explore the role of the patient's depressive symptom mood state on those associations.

**Methods:**

Self-report questionnaires on pain intensity, disability, relationship quality, perceived partner reactions to pain, and depressive symptoms were collected from participants (N = 174) taking part in a longitudinal study on low back pain within a primary care sample.

**Results:**

Participants reporting more consensus (e.g., agreement about sexual intimacy, level of affection) in their relationships had significantly higher pain intensity (*P* = 0.03), and solicitous partner responses (*P* = 0.04) were significantly positively associated with disability levels. However, the findings for pain intensity were only present in those with higher levels of depression, while the association of solicitous responses with disability was only significant in those with lower levels of depression, indicating a suppression effect of depression on pain and disability.

**Conclusions:**

Depressive symptoms play a significant role in determining the associations between relationship quality, perceived partner reactions, and pain and disability. The relationship construct of consensus and perceived solicitous responses were associated with pain and disability. These findings illustrate the importance of social context and patient mood state on the outcomes for those with low back pain.

## Introduction

Back pain is very common in the general population. A recent large-scale survey on back pain reports a lifetime prevalence of low back pain (LBP) of 57%, with up to 50% of back pain sufferers seeking health care in relation to their pain [1]. Many people with back pain will have limitations in daily life associated with their pain, and two-thirds are likely to have recurrent symptoms [2]. Back pain creates substantial health care costs, both direct (e.g., treatment) and indirect (e.g., informal care, loss of earnings, formal care, and provision of state support) for the individual, health care, and society [3,4].

It has been broadly accepted that the processes involved in the development, prognosis, and treatment of back pain are complex and fall under the auspices of the biopsychosocial model [5–9]. This model considers physiological pathology, individual variation in the experience of pain, individual variation in the management and coping related to pain, and the social context in which pain is experienced [10]. Since the introduction of this model, there has been a widespread application within research as well as implementation in treatment guidelines for back pain [11,12].

One area of research within the “social” component of the biopsychosocial model is the influence of the patient's immediate social context, for instance, the patients' spouse or partner. Research, largely on severe chronic pain samples in specialist clinic settings, has shown that pain can have negative effects on the satisfaction level or relationship quality between a person in pain and their partner [13,14]. In contrast, partners of those with pain can have direct effects on the way a person experiences and copes with pain [15–18]. There is also evidence that partner reactions related to the person's pain can have an impact on the person in pain's emotional well-being, for example, research has shown higher levels of depression in pain patients is associated with a higher level of negative partner responses [19]. Conversely, where partners are deemed too helpful or sympathetic (e.g., solicitous partner behavior), this may lead to an increase in reports of pain and disability in the patient [20,21]. In a recent review [22] of chronic pain in a “couples” context, there was a call for greater understanding of the complexities involved between persons with pain and their partners. Many studies have found links between pain severity and partner variables such as relationship satisfaction, negative and positive partner responses [23–27], but the evidence on direction and strength of associations is mixed, and further research is needed. Leonard et al.'s review [22] and other authors [20,28] state that more knowledge is required beyond a global focus on relationship quality, with further exploration of the wider constructs of relationship quality, such as communication between couples, levels of affection, and their level of argumentativeness.

**Figure 1:**
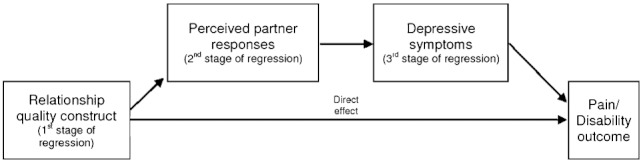
Association between relationship quality constructs and pain and disability outcome.

We are unaware of any research that has considered which constructs of relationship quality are most strongly associated with pain and disability. This is despite evidence to suggest there are clear conceptual differences in relationship quality that could be important for interventions for couples. For example, a recent review [29] of interventions for couples, where one person has a chronic illness, conclude that there is a further need to know about issues such as conflict, quality, communication, empathy, and satisfaction within the relationship rather than just focus on the spouse response to pain (i.e., operant effects). Furthermore, there is also the need to consider the role of affective components such as depression on relationship quality and pain. Prigerson et al. [30] showed that poor relationship quality is related to increases in depression and poor health outcomes. Tilden et al. [31] showed that the improvement of relationship quality can reduce depression. In relation to chronic pain, the Leonard et al. review [22] calls for a greater understanding of the role of patient mood state on relationship outcome, pain, and disability. The review concluded that depression is positively associated with pain and disability, marital satisfaction is negatively related to depression and, overall, populations with both pain and depression report lower levels of marital satisfaction than those with just pain alone. In this regard, we hypothesized that there will be a negative association between the constructs of relationship quality and pain and disability outcome. Some of this association will be explained by perceived partner negative and positive responses to pain and disability, and that the negative association between the constructs of relationship quality and pain and disability will increase in the presence of patient depressive symptoms (Figure 1). From this analysis, it will then be possible to identify how the various relationship constructs differ in their association with pain and disability, which may be helpful for those involved in the development of therapies for couples where one person has chronic pain.

The aims of this study are as follows: 1) to investigate the associations of key constructs of relationship quality (cohesion, consensus, and satisfaction) and perceived partner responses to pain behavior (e.g., solicitous and negative responses) with levels of pain and disability in those who reported LBP and 2) to explore the role of the patient's depressive symptom mood state on the associations between relationship quality variables and pain and disability.

## Methods

### Procedure

Participants were contacted as part of an ongoing longitudinal cohort study [32]. Participants in the original study were contacted by post following a primary care consultation for LBP in 2001–2002, and followed-up using postal self-completion questionnaires. For this current cross-sectional study, only those participants who had given informed consent, returned a follow-up questionnaire in 2009 (236 returned of 337 mailed, 70%), and signified that they had a current partner (either married or cohabiting) were included (N = 174). Participants were asked to fill out a questionnaire about their current level of pain and disability as well as measures of their relationship quality and mood. Participants were included even if they reported no pain or disability at this follow-up point in order to be reflective of a primary care population sample of previous back pain consulters and hence have a history of back pain, to provide a spectrum of the experiences of those who have reported and may currently report back pain, and to move beyond previous research focus on pain clinic populations or media recruitment methods [15,^16^,^19^,^21^,^24^,25].

Ethical approval for the study was granted by South Staffordshire Local Research Ethical Committee.

### Measures

Pain intensity was measured using the mean of three 0- to 10-point numerical rating scales (10 indicating worst pain) measuring least and average pain over the previous 2 weeks, plus current pain intensity [33]. Disability was assessed using the 23-item Roland Morris Disability Questionnaire (RMDQ) [34,35]. This gives a score from 0 to 23 (a higher score indicates a higher level of disability).

The Hospital Anxiety and Depression Scale (HADS) was used to measure depressive symptoms [36]. This consists of seven questions relating to depression on a scale from 0 to 21, with higher scores indicating higher levels of depression. It has been shown to be applicable to nonclinical samples [37].

The definition of having a partner in this study was “Your husband or your wife or the person that you live with” and this was assessed by a yes/no response. Relationship quality was assessed using the Revised Dyadic Adjustment Scale (RDAS) [38]. Responses can be scored in three subscales: cohesion (sharing ideas, working on things together, level of discussion and communication, and engagement of outside activities; range 0–19), consensus (agreement on sex relations, affection, religion, and major decisions; range 0–30), and satisfaction (level of disagreements and quarrelling, arguments, and thoughts on separation; range 0–20). A higher score indicates greater levels of these constructs between couples. In the original study [38], validity was shown to be good with a Cronbach's α of (0.9), split half testing (0.94). Subsequent studies have shown the RDAS to be able to discriminate between distressed and non-distressed couples [39], and versions of the measure has been validated within chronic back pain populations [19].

The West Haven-Yale Multidimensional Pain Inventory, Section 2 (MPI) [40], was used to measure perceived partner responses as rated by the patient. The MPI assesses the level of solicitous, negative and distracting behavior associated with the patients' partner when interacting with the patient in times of pain. The measure has 14 questions using a 6-point response scale scored on three subscales; negative response (range 0–6), solicitous response (range 0–6), and distracting response (range 0–6). The MPI has been used extensively within pain populations with reports of good reliability and reported population norms [14,^23^,^40^,41]. Only the solicitous and negative responses scales will be used in the analysis due to the lack of reported associations for the construct of “distraction” with pain and disability from previous research studies [22].

### Statistical Approach

Pearson's correlations were first used to assess associations between the relationship variables (RDAS and MPI subscales) and patient depressive symptoms, with pain and disability. Separate multiple linear regression models were then used to test the associations of each of the three RDAS relationship constructs (consensus, cohesion, and satisfaction) on 1) pain intensity and 2) disability. A three-stage approach was used to assess the associations between the constructs of relationship quality and pain and disability as well as to assess the change in explained variance with the addition of perceived partner responses and finally depressive symptoms. First, adjusting the association of relationship constructs with pain and disability only for age and gender. Then, adjusting further for perceived partner responses, and, finally, adjusting for patient depressive symptoms. This staged approach was chosen to control for the confounding effects of age and gender as reported in previous literature [22], to consider the extent of the association of relationship constructs with pain or disability when mediated by reported partner reactions (both solicitous and negative). Final adjustment for depressive symptoms was included due to the reported effects of depression both on relationship quality and pain and disability outcomes [19,30]. The *R*^2^ change statistic was reported to illustrate the change in overall variance explanation at each stage (e.g., stage 1—relationship construct, stage 2—perceived partner responses, stage 3—depressive symptoms) of each model, and unstandardized β values (regression coefficients) with 95% confidence intervals and standardized β were reported for each factor at the final stage of each model. Final model *F*-tests and adjusted *R*^2^ were reported. The assumptions relevant to multiple regression analysis were checked (e.g., linearity, multicollinearity, outlier leverage, constant variance, and residual normality) for all models and found to be satisfactory. Complete case analysis was performed for all models. Analysis was performed using SPSS, version 15 (SPSS, Inc., Chicago, IL, USA).

## Results

Comparisons with patients who consented to follow up in the original study in 2001 but either did not respond at this current follow-up stage or were not included in this study due to not having a current partner (N = 163) and those selected for inclusion within this study (N = 174) were carried out. Patients in this study, at original baseline, were no different in age or on reported pain intensity score but did report significantly less depressive symptoms (mean score 6.3 compared with 7.9), less disability (mean score 8.4 compared with 10.04), and include a greater proportion of females (61% compared with 53%).

Characteristics of responders to this current study are given in Table 1.

**Table 1:** Characteristics of study participants

	N	Range	Mean	SD	Median	IQR
Age (years)	174	37–67	54.33	7.97	55.5	14
Gender	174	Female 61%				
Length of relationship (years)	172	0–53	29.24	12.36	31	19
Pain intensity	174	0–10	2.73	2.77	1.67	3.67
RMDQ	174	0–23	5.35	6.02	3	6
Depression	173	0–20	4.59	4.03	3	6
*RDAS*						
Cohesion	172	0–19	10.87	3.79	11	5
Consensus	159	5–25	23.67	4.25	24	4
Satisfaction	171	4–20	14.47	2.81	14.5	3
*MPI*						
Negative	157	0–5	0.99	1.13	0.75	1.75
Solicitous	160	0–6	3.87	1.42	4	2

IQR = interquartile range; MPI = West Haven-Yale Multidimensional Pain Inventory; RDAS = Revised Dyadic Adjustment Scale; RMDQ = Roland Morris Disability Questionnaire; SD = standard deviation.

Missing data were apparent for consensus (8.9%), negative partner responses (9.8%), and solicitous partner responses (8.0%). For consensus, the only significant difference between item responders and nonresponders was that item nonresponders reported significantly lower levels of negative partners responses (*P* = 0.02). Non-item responders to partner negative reactions reported significantly greater levels of solicitous responses from their partners (*P* = 0.006) and those who did not respond to the solicitous behavior questions were significantly older (*P* = 0.027). Due to these missing data, the complete case analyses were on slightly reduced samples; cohesion (N = 148), consensus (N = 141), and satisfaction (N = 147) for both the pain and the disability models.

Females comprised 61% of the sample. The mean age of the sample was 54.33 years (standard deviation [SD] 7.97) and the mean time in their current relationship was 29.2 years (SD 12.4). Twenty-three percent of the cohort reported a score of 0 on the RMDQ and 22% reported a score of 0 on pain intensity. On questioning participants “How long since you had a whole month without back pain,” 65% responded that they had not had a month without back pain for over 3 months or more (20% responded no pain free period of a month in over 10 years).

Table 2 shows the correlations between the measures used in the study. Results show that depressive symptoms had a positive correlation with pain intensity and disability. This indicates that those who report greater depressive symptoms also report greater pain intensity and disability. There were no significant associations present for any relationship quality construct with pain intensity. However, those who reported lower cohesion (*r* = −0.156) or more negative responses (*r* = 0.162) between themselves and their partners reported increased disability, albeit with lower levels of association.

**Table 2:** Correlations between pain, disability, relationship quality, and depressive symptoms

Measure	Pain Intensity	RMDQ	Depression	Cohesion	Consensus	Satisfaction	Negative Responses	Solicitous Responses
Pain intensity	—							
RMDQ	0.720**							
Depression	0.359**	0.492**						
Cohesion	−0.058	−0.156*	−0.294**					
Consensus	0.016	−0.045	−0.300**	0.575**				
Satisfaction	−0.027	−0.075	−0.266**	0.630**	0.704**			
Negative responses	0.072	0.162*	0.274**	−0.422**	−0.508**	−0.470**		
Solicitous responses	−0.016	0.073	−0.088	0.411**	0.355**	0.353**	−0.311**	—

* Correlation significant at the 0.05 level (two-tailed)

** Correlation significant at the 0.01 level (two-tailed).

RMDQ = Roland Morris Disability Questionnaire.

### Associations with Pain Intensity

At stage 1, there was no identified association between the relationship constructs and pain intensity. Similarly, there were no associations at stage 2 when the perceived partner response variables were added to the models. However, with the addition of depressive symptoms at stage 3, depressive symptoms (*P* < 0.001) and consensus (*P* = 0.035) were independently positively related to pain intensity (Table 3). This indicated that those who reported more depressive symptoms and more consensus between themselves and their partner also reported greater pain intensity. Overall, each model accounted for between 8% and 10% of the variance in pain intensity scores. There was no effect found for age or gender at any stage of the analysis (initial model with only age and gender, *R*^2^ = 0.007).

**Table 3:** Multiple regression model of associations of relationship quality with participant pain intensity

Model†	Variable	Final Model β (95% Confidence Interval)	Final Model Standardized β	Model Tests
Cohesion	Age	0.002 (−0.048, 0.052)	0.006	*F*_(6,148)_ 3.47, *P* = 0.003 (*R*^2^ adjusted = 0.088)
	Gender	0.340 (−0.53, 1.21)	0.063	
	Cohesion	0.030 (−0.09, 0.158)	0.043	
	Negative	0.019 (−0.401, 0.439)	0.008	
	Solicitous	−0.019 (−0.346, 0.307)	−0.010	
	Depression	0.223 (0.119, 0.327)	0.347**	
Consensus	Age	0.003 (−0.047, 0.052)	0.008	*F*_(6,141)_ 3.68, *P* = 0.002 (*R*^2^ adjusted = 0.099)
	Gender	0.293 (−0.587, 1.17)	0.055	
	Consensus	0.123 (0.009, 0.237)	0.203*	
	Negative	0.157 (−0.282, 0.597)	0.070	
	Solicitous	−0.066 (−0.377, 0.246)	−0.036	
	Depression	0.226 (0.121, 0.331)**	0.354**	
Satisfaction	Age	−0.004 (−0.055, 0.046)	−0.013	*F*_(6,147)_ 3.89, *P* = 0.001 (*R*^2^ adjusted = 0.102)
	Gender	0.460 (−0.415, 1.33)	0.085	
	Satisfaction	0.150 (−0.021, 0.322)	0.161	
	Negative	0.134 (−0.301, 0.569)	0.057	
	Solicitous	−0.065 (−0.383, 0.252)	−0.035	
	Depression	0.228 (0.125, 0.331)**	0.354**	

	*R*^2^ Change Statistics for Pain Analysis Models			
	
	Model	Stage 1 (Relationship Construct/Age/Gender) *R*^2^ Change	Stage 2 (as Stage 1 with Perceived Partner Responses) *R*^2^ Change	Stage 3 (as Stage 2 with Depressive Symptoms) *R*^2^ Change
	Cohesion	0.012	0.006	0.106
	Consensus	0.007	0.018	0.110
	Satisfaction	0.010	0.015	0.112

* *P* value < 0.05

** *P* value < 0.001

† Adjustment for age and gender was carried out at all stages in all models.

### Associations with Disability

At stage 1, there were no significant associations between the relationship constructs and disability. At stage 2, perceived negative responses within all models and solicitous within the cohesion model were significant. On the addition of depressive symptoms at stage 3, greater depressive symptoms (*P* < 0.001) and greater solicitous behavior (*P* = 0.044, within the cohesion model) were significantly associated with higher disability (Table 4). Overall, each model accounted for between 21% and 24% of the variance in disability scores. There was no effect found for age or gender at any stage of the analysis (initial model with only age and gender adjusted *R*^2^ = −0.009).

**Table 4:** Multiple regression model of association of relationship quality with participant disability

Model†	Variable	Final Model β (95% Confidence Interval)	Final Model Standardized β	Model Tests
Cohesion	Age	−0.006 (−0.109, 0.096)	−0.009	*F*_(6,148)_ 8.17, *P* < 0.001 (*R*^2^ adjusted = 0.218)
	Gender	0.826 (−0.955, 2.61)	0.069	
	Cohesion	−0.071 (−0.333, 0.191)	−0.046	
	Negative	0.428 (−0.430, 1.28)	0.083	
	Solicitous	0.684 (0.17, 1.35)	0.165*	
	Depression	0.641 (0.428, 0.854)	0.451**	
Consensus	Age	−0.009 (−0.111, 0.093)	−0.012	*F*_(6,141)_ 8.59, *P* < 0.001 (*R*^2^ adjusted = 0.237)
	Gender	0.808 (−1.01, 2.63)	0.068	
	Consensus	0.224 (−0.012, 0.461)	0.165	
	Negative	0.766 (−0.143, 1.68)	0.151	
	Solicitous	0.537 (−0.107, 1.81)	0.132	
	Depression	0.706 (0.488, 0.923)	0.492**	
Satisfaction	Age	−0.015 (−0.119, 0.089)	−0.021	*F*_(6,147)_ 8.105, *P* < 0.001 (*R*^2^ adjusted = 0.218)
	Gender	0.979 (−0.826, 2.78)	0.082	
	Satisfaction	0.173 (−0.181, 0.526)	0.084	
	Negative	0.671 (−0.225, 1.56)	0.130	
	Solicitous	0.558 (−0.097, 1.21)	0.134	
	Depression	0.663 (0.451, 0.876)	0.466**	

	*R*^2^ Change Statistics for Disability Analysis Models			
	
	Model	Stage 1 (Relationship Construct/Age/Gender) *R*^2^ Change	Stage 2 (as Stage 1 with Perceived Partner Responses) *R*^2^ Change	Stage 3 (as Stage 2 with Depressive Symptoms) *R*^2^ Change
	Cohesion	0.023	0.046	0.180	
	Consensus	0.002	0.053	0.213	
	Satisfaction	0.009	0.045	0.194	

* *P* value < 0.05

** *P* value < 0.001

† Adjustment for age and gender was carried out at all stages in all models.

In light of the suppression effect of depression leading to a positive association between consensus and pain and disability, multiple regression models stratifying for patient depressive symptoms were carried out to investigate further the influence of depression on the association of relationship constructs with pain and disability. Stratification was carried out on patient depressive symptoms using a cut-off score on the HADS based on the mean score (3.68) of a normative population sample [37]. Patient depressive symptoms scores were stratified to create low (N = 89) and high (N = 84) depressive symptom groups (Table 5). Multiple regression models were run to test for significant effects in a similar way to the full models described earlier. Among the high depressive symptom group, only consensus was associated with pain intensity (*P* = 0.047), with those who reported higher consensus between themselves and their partner, also reporting higher pain intensity. There were no associations between the relationship quality variables and pain intensity for those within the low depressive symptom group. Higher perceived solicitous responses were associated with higher disability levels in all of the relationship quality models (*P* = 0.036, cohesion; *P* = 0.032, consensus; *P* = 0.031, satisfaction) within the low depressive symptom group (Table 5). Overall *F*-tests were not significant for the models within the stratified analysis.

**Table 5:** Associations of relationship quality constructs with pain and disability for participants with high and low depressive symptoms

	Relationship Construct	Variable	Low Depression Group β (95% CI) (Standardized β) (N = 89)	High Depression Group β (95% CI) (Standardized β) (N = 84)
Pain intensity model	Cohesion	Cohesion	0.076 (−0.076, 0.228)	−0.015 (−0.240, 0.210)
		Negative responses	−0.159 (−0.661, 0.342)	0.291 (−0.420, 1.002)
		Solicitous responses	0.057 (−0.301, 0.415)	−0.184 (−0.813, 0.446)
	Consensus	Consensus	0.091 (−0.088, 0.270)	0.162 (0.002, 0.321) (0.296)*
		Negative responses	−0.109 (−0.684, 0.466)	0.566 (−0.115, 1.25)
		Solicitous responses	0.071 (−0.280, 0.422)	−0.341 (−0.925, 0.243)
	Satisfaction	Satisfaction	0.115 (−0.104, 0.334)	0.192 (−0.082, 0.467)
		Negative responses	−0.139 (−0.668, 0.389)	0.504 (−0.210, 1.22)
		Solicitous responses	0.071 (−0.273, 0.416)	−0.318 (−0.942, 0.306)
Disability model	Cohesion	Cohesion	0.057 (−0.155, 0.270)	−0.192 (−0.738, 0.353)
		Negative responses	0.212 (−0.489, 0.912)	1.18 (−0.545, 2.904)
		Solicitous responses	0.538 (0.037, 1.038) (0.255)*	0.820 (−0.707, 2.34)
	Consensus	Consensus	0.163 (−0.084, 0.409)	0.260 (−0.151, 0.671)
		Negative responses	0.444 (−0.348, 1.24)	1.745 (−0.01, 3.501)
		Solicitous responses	0.529 (0.046, 1.01) (0.250)*	0.443 (−0.106, 1.95)
	Satisfaction	Satisfaction	0.138 (−0.168, 0.443)	0.158 (−0.519, .835)
		Negative responses	0.273 (−0.466, 1.01)	1.533 (−0.208, 3.32)
		Solicitous responses	0.533 (0.51, 1.02) (0.250)*	0.578 (−0.961, 2.12)

* *P* value 0.05, [β] = standardized β (only shown for significant results within the models).

CI = confidence interval.

## Discussion

This study shows that relationship quality constructs such as consensus, cohesion, and satisfaction have no direct association with pain intensity or disability in this cohort who had previously consulted for back pain. However, when participant depressive symptoms were entered into the regression models, aspects of relationship quality and reported partner responses became significantly associated with pain and disability.

It has been demonstrated previously that depression can act as a mediator between relationship quality and pain outcome [19]. This was not shown in this study because, according to Baron and Kenny's mediation model [42], there was no initial association between the predictor (relationship quality variables) and outcomes (pain/disability) for depressive symptoms to mediate. According to a model of MacKinnon et al. [43], the effects of depressive symptoms, rather than mediate, have a suppressive effect. This effect occurs when a controlled variable (in this case depressive symptoms) has a negative relationship with the independent variable (relationship quality) but a positive relationship with the dependent variable (pain/disability). Depressive symptoms thereby mask the influence of relationship quality on pain/disability, and when controlled, an association between some relationship quality variables with pain and disability is revealed. In confirmation, stratified analysis indicated that possible specific aspects of relationship quality, such as consensus between patient and partner, are related to pain intensity, and solicitous partner effects, perceived by the patient about their partner, may be associated with disability.

The finding that participants reported greater consensus with their partner (e.g., agreement about sexual relations, affection, and agreement about major decisions) as pain intensity increased, even in the presence of higher levels of depressive symptoms, appears counterintuitive. Evidence has shown that increases in psychological distress such as depression are associated with lower levels of reported relationship quality [44], and this study has demonstrated strong negative associations between relationship quality outcomes and depressive symptoms. One interpretation of this effect could be the “adaptation process” for couples, when one partner has a chronic illness. Studies have shown that couples with one of the partners having a disability, actually augment the perception of the quality of their relationship [45]. However, this process, where relationship quality may improve between couples, is usually attributed to those with long-term stable conditions, whereas those with short-term or intermittent illnesses tend to have the opposite effect of decreasing relationship quality [46]. It may be that couples within this study have adapted and accommodated to living with pain intensity due to the number of years with LBP (at least 7 years) and the significant time that they have been in their current relationship (mean 29 years). These findings suggest that couples may overcome and adapt to living with pain and disability in the long term. However, findings may well be different when people first encounter back pain, with evidence of potential consequences (e.g., losing one's job, changing roles within the family, disability) at the start of a person's LBP that may have an effect on the relationship quality between couples [47], which, in turn, may moderate the prognosis for the person with back pain.

We have shown that increases of perceived solicitous behavior from partners (e.g., getting the person in pain to rest, taking over their jobs and duties) are associated with increases in disability levels. According to theoretical models underpinning solicitous behavior [20,40], this association may confirm the presence of an operant effect. More interestingly, this association is found in all relationship quality models (consensus, cohesion, and satisfaction) for those with lower levels of depressive symptoms. However, some previous literature has reported opposing effects, whereby solicitous behavior is associated with physical dysfunction, but only in those with high depressive symptoms. For example, Romano et al. [26] showed, in their study of chronic pain patients, a positive association between nonverbal pain behavior displayed by the patients and actual solicitous behavior from their partners and this effect significantly increased if the patient also reported depressive symptoms. A similar conclusion was reached by a study on children's functional disability (Peterson and Palermo), whereby again the level of solicitous responses increased in children with higher levels of emotional distress [48]. The Peterson and Palermo and Romano et al. studies suggest that perhaps there is an additive effect, where distress elicits more solicitous responses in addition to disability. Romano et al. and Peterson and Palermo gathered information not only from the index patient but also from the provider of solicitous responses (e.g., spouse, parent, and caregiver), and indeed, when Romano et al. examined only patient reported data the effect of increased depression leading, to a greater association between solicitous behavior and functional outcome disappeared. It may be the depressed patient is less likely to interpret and report their partner's behavior in a positive light (i.e., being solicitous) and so the collection of only patient information about partnerships may be prone to different influences based on the patient level of pain or depression [23]. However, although the Romano et al. study reports similar overall relationship quality scores (comparing the Dyadic Adjustment Scale scores to the revised version used in this study), they report a less average length of relationship (12 years to this study's 29 years), have the possibility of greater severity of pain (patients were entered in a pain treatment program with over 60% receiving disability benefits), and their cohort of patients report greater levels of depression (this current study reports a similar median score compared with population norms for depression [37]), whereas Romano et al. reported depression levels at twice population norms [49]. Alternatively, our findings could suggest a dual role for solicitous behavior. Two previous studies have shown that partner solicitous behavior may act as a buffer against patient depression in those with chronic pain [50,51]. This study found an association of solicitous responses with disability in those who reported low levels of depressive symptoms with no association for those with high levels of depressive symptoms, and given that the correlation results show that higher disability is strongly associated with increases in depressive symptoms (*r* = 0.492), there may be a buffering effect at work. Therefore, the solicitous partner may reduce depression for those with high levels of disability due to their solicitous behavior. This raises the issue of a possible dual role for solicitous behavior from partners, one being a barrier to recovery by increasing disability (operant effect) but secondly facilitating recovery by reducing depression. Further longitudinal research is clearly needed to understand the complex interaction effects of solicitous behavior over time, e.g., to establish if possible short-term benefits (i.e., reduction in depression caused by the pain/disability) may transform into long-term harms (i.e., operant effects leading to maintenance or increase in disability).

There are limitations to this study that require consideration. The study's cross-sectional design does not tell us the direction effect of the variables and we cannot make assumptions on causality. Indeed, the precise temporal role for all of these factors is for debate, with many competing models [19,^20^,^22^,23] placing depression, relationship quality, partner responses, and pain/disability at hypothesized causal points. The results show that there were significant differences between those who responded and were eligible to be included (i.e., having a current partner) and those who did not in this study. Those included reported significantly lower levels of depressive symptoms and lower levels of disability at the original study baseline and were also more likely to be female. Furthermore, some questions about the person's relationship quality incurred lower responses within this study. Considering the reasons for non-item response for the particular questions (consensus, negative and solicitous responses), it is clear that those questions touch on aspects about the relationship between the participants and their partner and furthermore ask about aspects of that relationship that may be sensitive (e.g., sexual satisfaction, arguments, and negative responses from partners). It is not uncommon for questions such as these to incur lower levels of response than non-invasive questions [52,53]. However, missing responses to specific sensitive questions may indicate a possible overall reluctance to “tell it like it is,” be that whether a partner is perceived to be overly critical or overly solicitous. Connected to this is the use of the RDAS measure. The RDAS is a validated measure of the relationship quality between couples and was chosen because of its validity in pain populations and ease of use for participants. However, further research will be needed to explore the clinical significance and meaning of this study's findings and how the RDAS constructs can translate into actual interactions between couples.

Another limitation is that the sample only represents those who have had LBP in the long term with some people currently reporting no or little pain or disability, and so may not be applicable to LBP patients within the acute phases. This may have had an effect on the perceived reactions from partners. The measures on perceived reactions from partners are specifically targeted to ascertain information about when the patient is in pain and the reactions from spouses to this pain, and as such, could be subject to bias in this study, where a participant retrospectively recalls a time when they are in pain (recall bias) or where they have no comparison at that time (i.e., no pain). This may lessen the sensitivity to pick up on perceptions of negative or solicitous behavior from spouses. We had very similar scores of the solicitous responses to normative values reported by Nicholas et al. [41] (3.54 vs 3.87) but the level of negative partner responses is lower (3.54 vs 0.99) and this may be reflective of having an overall lower level of pain and disability within the cohort. However, this study's cohort consists of patients mostly with a long history of back pain, some of whom may have recovered, or are outside of an episode, with 65% of the cohort indicating not having a month free of back pain for over 3 months (20% indicated continuous pain for over 10 years). We believe the diverse range of pain and disability severity in this cohort gives a view of patient and partner function beyond more severe pain cohorts seen in secondary care clinics or more severely chronic pain populations where much previous research has focused.

Finally, although the assumptions for the multiple regression analysis were checked and found to be adequate, the additional further analysis reported in this article, although producing findings of interest, is tempered by lower sample sizes for the stratified groups. In addition, the inclusion of the stratified analysis also increased the chance of a type 1 error by increased multiple testing, and therefore, further independent research is required to investigate the complex interactions between depression, relationship quality, and pain.

## Conclusions

Overall, the results of this study illustrate that relationship quality variables are associated with patient levels of pain and disability, but this appears to be dependent on the patient's level of depressive symptoms. Similarly, some associations between reported partner responses are dependent on the constructs of relationship quality, but again appear dependent on the patient's level of depressive symptoms. Further analysis has shown that there are associations of perceived solicitous effects with disability for those with low levels of depressive symptoms, which may indicate positive reinforcement but also may indicate a buffer to patient depressive symptoms. This study, and other previous findings, demonstrates partner context effects on patient pain and disability. Further longitudinal investigation is needed to establish the direction of these effects and the possibility of utilizing this information in the formulation of treatments for those with LBP that are inclusive of social factors. Researchers have previously recommended treatments that include consideration of marital therapy to reduce negative effects from partners [23]. We have demonstrated that partner effects are present, through relationship quality, that may show a positive effect (e.g., buffer from depression) or a maladaptive effect (e.g., solicitous effect). These findings should be further investigated, with consideration given to possible improvements of LBP interventions.
